# Adolescent Connectedness with Parents Promotes Resilience among Homeless Youth

**DOI:** 10.3390/children5070096

**Published:** 2018-07-16

**Authors:** Kristen M. Aggerbeck Kessler, Debanjana Chatterjee, Rebecca Shlafer, Andrew J. Barnes

**Affiliations:** 1 Developmental-Behavioral Pediatrics, Division of General Pediatrics and Adolescent Health, Department of Pediatrics, University of Minnesota Medical School, Minneapolis, MN 55455, USA; 2 Division of General Pediatrics and Adolescent Health, Department of Pediatrics, University of Minnesota Medical School, Minneapolis, MN 55455, USA; debanjana.chatterjee@gmail.com (D.C.); shlaf002@umn.edu (R.S.)

**Keywords:** adolescents, homeless, resilience, parents, internal assets

## Abstract

Youth who experience homelessness have worse health and well-being than housed youth. Internal assets, including social competency and positive self-identity, are factors that promote healthy development. This study compared internal assets between homeless and housed youth, and examined whether connectedness with parents moderates the association between homelessness and internal assets. Using data from a large population-based survey of middle- and high-school aged youth, we found that homelessness was associated with lower levels of internal assets. However, having high connectedness with a parent significantly predicted the strength of these assets, suggesting opportunities to promote health equity among homeless youth.

## 1. Introduction

Nearly 2.5 million children are homeless in the United States (U.S.), representing 1 in 30 U.S. children [1]. Research has shown that homelessness is associated with worse well-being among youth [1,2,3,4,5,6]. Homeless youth experience higher rates of health problems than their non-homeless peers [7]. Chronic health conditions, including asthma and mental health problems, are highly prevalent among these youth [8,9,10]. In addition, the stress that often accompanies homelessness can change the brain’s architecture and function, interfering with learning, cognition, social skills, executive functioning, and emotional self-regulation [1,11,12]. This goes beyond the effects of poverty, with homeless youth experiencing greater risk than impoverished-but-housed youth [2,4,13]. Despite these complex risks, there is also great variability in outcomes among homeless youth, demonstrating important capacity for positive adaptation, also known as resilience [1,2,14,15].

Developmental assets are the essential resources and supports that a young person needs to flourish and be healthy [16,17,18]. Internal assets, including social competency and positive self-identity, are skills and self-perceptions that guide young people to become self-regulating [16]. The developmental asset framework has been validated across race/ethnicity, gender, socioeconomic background, and geographic location [17]. Research has shown that possessing developmental assets helps not only to prevent high-risk behavior, but also to promote healthy development and resilience [16,17]. The more developmental assets a young person has, the less likely they are to engage in risk-taking behavior [16]. This includes being less likely to engage in violence, drug and alcohol use, delinquency, and early sexual intercourse [16]. This is true for high-risk youth as well. In fact, research demonstrates that vulnerable youth, for example those who experience poverty, violence, or abuse, often experience a greater protective benefit from developmental assets than youth without such vulnerabilities [16]. In addition to reducing risky behavior, having developmental assets promotes positive outcomes, including better health and school functioning [16,18].

One such developmental asset is connectedness, which refers to the positive relationships an adolescent has with individuals in their lives. A strong relationship with a parent promotes healthy development. In fact, Blum and Blum described family connectedness as “the single most protective factor reducing negative outcomes for young people” [19] (p. 56). Prior studies have shown that a strong relationship with a parent reduces risk-taking behavior including alcohol, marijuana and tobacco use, suicidal ideation and attempts, violence, and early age of sexual initiation [20,21,22,23,24]. A strong connection with a parent is also associated with more positive self-reported outcomes including high self-worth, less emotional distress, and better overall health [20,21].

The links between the stress of homelessness, health disparities between homeless and housed youth, and negative outcomes among homeless youth are well-established [2,3,4,7,9,10,25]. Additionally, connectedness with a parent during adolescence can reduce health risk by promoting resilience [20,22,26]. To date, research has not examined internal assets or connectedness among homeless youth specifically. Similarly, no studies have considered the role of parent connectedness on the development of internal assets among homeless youth, which could reduce health disparities by promoting resilience in this population.

This study had two main objectives. First, we aimed to replicate previous findings about protective factors among housed youth and compare the associations between homeless vs. housed youths’ reports of their internal assets [20,22,26,27]. We then investigated whether youths’ connectedness with parents modified the association between homelessness and youths’ internal assets.

## 2. Materials and Methods

### 2.1. Participants

Data were obtained from the Minnesota Student Survey (MSS) [28]. The survey is conducted every three years through partnerships between the Minnesota Departments of Education, Health, and Public Safety and focuses on a variety of risk and protective factors that impact the lives of young people. In 2013, 84% of public school districts in Minnesota participated and overall student participation was 67%. The survey includes 5th, 8th, 9th, and 11th graders. For this analysis, data from 9th and 11th graders from public and charter schools were included (*n* = 79,339). The University of Minnesota Institutional Review Board considered this study exempt from IRB review because the data were completely anonymous.

### 2.2. Measures

#### 2.2.1. Homelessness

Students responded to a single question regarding housing status, “During the past 12 months, have you stayed in a shelter, somewhere not intended as a place to live, or someone else’s home because you had no other place to stay.” Responses were “No”, “Yes—I was with my parents or an adult family member”, or “Yes—I was on my own without any adult family members”. The homeless variable was dichotomized to “1 = yes” (ever homeless in the last year, with or without a family member) or “0 = no” (never homeless in the last year).

#### 2.2.2. Internal Assets

The primary independent variable was internal assets, which was assessed using 14 items from the Developmental Assets Profile [29]. Complete subscales for two of the internal assets, positive identity and social competency were included in the MSS. There were six items (α = 0.82) on positive identity, including “I feel in control of my life and future”, “I am thinking about what my purpose is in life”, and “I find good ways to deal with things that are hard in my life”. There were eight items (α = 0.84) on social competency, including “I plan ahead and make good choices”, “I say no to things that are dangerous or unhealthy”, and “I express my feelings in proper ways”. Students responded on a four-point scale from “not at all or rarely” to “extremely or almost always”. These 14 items were averaged to create a composite measure of internal assets (Range = 1–4; α = 0.90).

#### 2.2.3. Adolescent–Parent Connectedness

Youth responded to three questions about their relationships with parents. Questions included “Can you talk to your father about problems you are having?” and “Can you talk to your mother about problems you are having?” The five responses were “Yes, most of the time”, “Yes, some of the time”, “No, not very often”, “No, not at all”, and “My father/mother is not around”. They also completed the question, “How much do you feel your parents care about you?” Responses were on a four-point scale from “not at all” to “very much”. Students were considered to have a strong parent connection if they answered yes to either of the questions regarding if they could talk to one of their parents and felt their parents cared about them “quite a bit” or “very much”. We then dichotomized this variable into high and low parent connection [30].

### 2.3. Statistical Analysis

Analyses were conducted using SAS 9.4 (2014). In our sample of 79,339 of 9th and 11th graders, 7.3% had missing information on status of homelessness, 1.1% on race and ethnicity, 2.5% on the poverty measure, 0.3% on parent-adolescent connectedness, and less than 1% for parent communication and household composition. For internal assets, we did not have information on 5.8% of the students in our sample because of non-response to at least 11 items in the questionnaire used to create the internal assets score.

To determine the impact of adolescent–parent connectedness on internal assets among homeless youth, a univariate model first examined the association between homelessness and internal assets. Multiple linear regression models then tested the association between homelessness and internal assets, controlling for key covariates (i.e., youth race, gender, grade, school location, free/reduced price lunch, and family structure). A final model included interaction terms to test hypotheses regarding moderation of internal assets by connectedness with parents. Significance values of *p* < 0.05 were considered statistically significant.

## 3. Results

### 3.1. Prevalence of Homelessness

Five percent (*n* = 3627) of Minnesota high school students reported being homeless in the past year. Of those who were homeless, 2.8% (*n* = 106) reported that they were homeless without an adult relative. A history of homelessness in the last year was significantly more prevalent among non-white and Hispanic students, males, ninth grade students, those who reported receiving free/reduced price lunch or attending school in a non-metro area, and students living in a family structure other than a two-parent household (Table 1).

### 3.2. Internal Assets and Homelessness

The internal assets score for the overall sample ranged from 1–4 (Mean 3.0 [*SD*] = 0.58). The mean score for internal assets was significantly lower among those who were homeless (2.64) compared to those who were not homeless (2.99; *p* < 0.001) (Table 1). After adjusting for covariates including race, gender, grade, school location, free lunch and family structure, being homeless in the last year was significantly negatively associated with internal assets. On average, homeless youth reported an internal assets score of 0.28 units lower than housed youth.

### 3.3. Parent Connection as a Moderator

**Figure 1:**
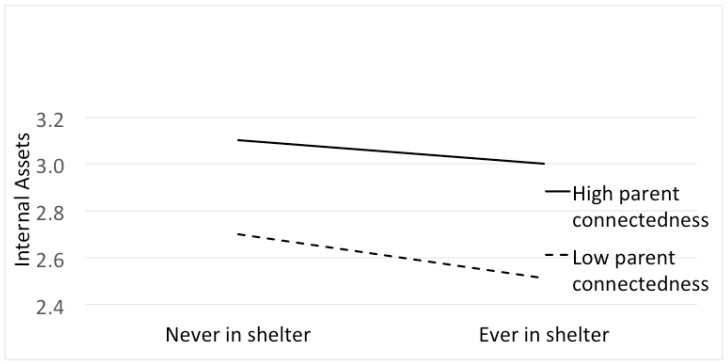
Internal assets, by parent connectedness, for homeless vs. housed youth.

Among homeless youth, 42% reported a high connection with a parent compared to 68% of non-homeless youth (*p* < 0.0001). Consistent with our hypothesis, the association between homelessness and internal assets was moderated by parent connectedness (Table 2). Based on the results of this model and the significance of the interaction term, we calculated the differences in internal asset among homelessness and non-homeless children classified by high and low parent connectedness. Compared to non-homeless youth, homeless youth who had a high parent connection had a decline in internal assets of only 0.13 units compared to a decline of 0.24 units among homeless youth with a low parent connection (interaction *p* < 0.001) (Figure 1).

## 4. Discussion

Of the youth surveyed in Minnesota, nearly 5% reported being homeless in the last year. This was higher than the national estimate of about 3% [1]. Homelessness was more prevalent among non-white and Hispanic students, males, ninth grade students, those with access to free lunch, those living in non-metro areas and students living in a family structure other than a two parent household. On average, youth who were homeless reported lower levels of internal assets compared to youth who were not homeless. In addition, homeless youth reported a lower connection with parents compared to non-homeless youth. These associations were significant even after accounting for race, gender, grade, school location, free lunch and family structure. Despite demonstrating that homeless youth have lower internal assets on average than housed youth, this study also demonstrates that high connectedness can partially ameliorate this disparity in internal assets. Homeless and housed youth who had a high connection with a parent had higher developmental assets than youth who reported a low parent connection.

Results of this study are consistent with prior studies on internal assets among homeless youth who were found to have fewer developmental assets, both internal and external, compared to non-homeless youth [27]. Furthermore, prior literature highlights how parent connection and support can mitigate some of the negative effects of traumatic experiences, including homelessness [13]. Parents who are more responsive to their child provide the child with a sense of self-efficacy, safety, and trust that promotes self-regulation [13]. Children who have positive co-regulation with parents are more capable of managing stress and navigating developmental tasks [13]. Such regulation of ones’ self is thus another example of an internal asset (not measured in this study) that is better under conditions of high parent support—a finding that is extended by the association found in this study between high parent connection and other internal assets (positive identity and social competency) among homeless and non-homeless youth alike.

Homeless youth are underrepresented in the literature in general, including the literature on positive youth development. Most studies of homeless youth have sampled only those who are unaccompanied rather than include homeless youth in families. Additionally, many of the studies focusing on homeless youth use a risk or deficit perspective, rather than focusing on factors that may increase resilience in this population. Although we found disparities in internal assets based on homeless status, we found that better connectedness with parents can reduce these disparities. Because internal assets help protect against negative health behaviors and health outcomes, reducing the gap in these assets between housed and homeless youth may lead to improved health equity for homeless youth. Nevertheless, we found that while connectedness with parents improves internal assets, it does not normalize them to the levels enjoyed by non-homeless youth. This implies that other supports (e.g., the provision of trauma-informed care) are likely needed to fully buffer the risk experienced by homeless adolescents.

While this study was based on a large sample size, it was a cross-sectional design and conclusions about the cause and effect relationship between homelessness and internal assets, as well as the effect of high parent connectedness, cannot be made. There was a small proportion of the sample that reported being homeless alone or unaccompanied. Given the limited number, these students were grouped with those who reported being homeless with an adult relative. However, youth who are homeless alone may have very different connections to adults and community resources than those who are with family. They may also be at a different level of risk for negative outcomes. Looking at unaccompanied homeless youth alone was outside the scope of the current study, but future research could examine this. Additionally, some youth do not attend school regularly and they would not have been included in this school-based survey. The survey measures were self-reported by youth and this may have prompted some inflation of responses regarding internal assets, causing concentration at the high end of the scale. Parent–adolescent connectedness is often measured by assessing the quality and characteristics of the relationship. In this study, the relationship was based on youths’ reports of feeling like their parent cares about them and if they can talk to their parent. These measures do not take into account the characteristics or quality of the relationship in detail. 

Despite the limitations, this study has important implications for policy and practice. Programs should be developed to increase the connection parents have with youth in general, but particularly among vulnerable homeless youth. Connectedness does not normalize internal assets to the rates seen in non-homeless youth. This demonstrates the complexity of the risks associated with homelessness and the severity of the disparities among homeless youth and the necessity for robust programming that targets not only connectedness, but perhaps also stable housing and psychosocial resources for these young people

## 5. Conclusions

Youth who experience homelessness are at increased risk for negative health, achievement, and developmental outcomes. Results of this study support the link between homelessness and decreased internal assets, as well as lower levels of connectedness with parents. However, this study also demonstrated a potential pathway of resilience. Positive connections promote internal assets that are critical for health and well-being among all youth. Further studies should investigate interventions that focus on strengthening the relationships youth have with parents, other adults, school, and community.

## Figures and Tables

**Table 1:** Sample characteristics: demographics, family composition, and socio-economic variables

	Total *n* = 79,339	Non-Homeless *n* = 69,933 (95.1%)	Homeless *n* = 3627 (4.9%)	*p*-Value
	% or Mean (SD)			
Race				<0.0001
American Indian (non-Hisp)	1.00	0.91	2.32	
Asian (non-Hisp)	5.63	5.15	8.37	
Black (non-Hisp)	5.14	4.24	7.36	
Other (non-Hisp)	0.23	0.19	0.59	
White (non-Hisp)	74.6	76.9	60.2	
Multi (non-Hisp)	6.54	6.23	10.6	
Hispanic	6.84	6.38	10.6	
Gender				<0.0001
Male	50.16	49.05	55.2	
Female	49.84	50.95	44.8	
Grade				<0.0001
9th	53.42	52.68	58.8	
11th	46.58	47.32	41.2	
Access to free lunch in school				<0.0001
Yes	26.29	24.11	46.0	
No	73.71	75.89	54.0	
School location				<0.0001
Twin cities metro area	46.75	48.23	48.9	
Greater Minnesota	53.25	51.77	51.1	
Family composition				<0.0001
Two-parent household	33.83	68.11	44.2	
Others	66.17	31.89	55.8	
Connectedness with parent				<0.0001
High	67.08	68.65	41.8	
Low	32.92	31.35	58.2	
Internal assets	2.96 (0.58)	2.99 (0.70)	2.64 (0.66)	<0.0001

**Table 2:** Multivariate model.

	Estimate	Standard Error	*p*-Value
Homelessness	−0.23	0.012	<0.0001
Parent connectedness	0.41	0.004	<0.0001
Homelessness*parent connectedness	0.10	0.019	<0.0001
American Indian (non-Hisp)	−0.10	0.022	<0.0001
Asian (non-Hisp)	0.09	0.012	<0.0001
Black (non-Hisp)	0.13	0.012	<0.0001
Other (non-Hisp)	−0.03	0.045	0.5431
White (non-Hisp)	0.06	0.008	<0.0001
Multi (non-Hisp)	0.002	0.011	0.8257
Received free or reduced-price lunch at school	0.08	0.005	<0.0001
Grade	−0.02	0.002	<0.0001
Gender (female vs. male)	0.01	0.004	0.0002
Lives in metro area	0.06	0.004	<0.0001
Lives in two-parent household	0.10	0.004	<0.0001
